# Web-based nutrition: a useful resource for cancer patients?

**DOI:** 10.3389/fnut.2023.1134793

**Published:** 2023-06-30

**Authors:** Diana Elena Lazar, Roxana Postolica, Bianca Hanganu, Veronica Mocanu, Beatrice Gabriela Ioan

**Affiliations:** ^1^Doctoral School, Grigore T. Popa University of Medicine and Pharmacy, Iași, Romania; ^2^Municipal Hospital “St. Hierarch Dr. Luca”, Onesti, Romania; ^3^Department of Psychology, Regional Institute of Oncology, Iași, Romania; ^4^IIIrd Medical Department, Legal Medicine, Faculty of Medicine, Grigore T. Popa University of Medicine and Pharmacy, Iași, Romania; ^5^Department of Morpho-Functional Sciences (Pathophysiology), Grigore T. Popa University of Medicine and Pharmacy, Iași, Romania

**Keywords:** cancer, web-based nutrition, dietary advice, cancer personalized nutrition, cachexia, sarcopenia, telemedicine

## Abstract

**Introduction:**

An accessible and affordable way to deliver behavior change programs to a large proportion of the growing community of cancer patients and survivors is through web-based methods of nutritional counselling.

**Objective:**

The aim of this systematic review was to evaluate the effectiveness of web-based nutritional therapies targeting physical activity, diet, and/or weight control for cancer patients or survivors, primarily disseminated via modern technologies (mobile phone applications) or online.

**Materials and methods:**

The authors conducted a structured search of the PubMed database. Studies that have focused on physical activity (PA) and dietary change and/or weight control in adolescent and adult cancer patients and survivors have reported outcomes conducted *via* a broad modality.

**Results:**

Nine articles focused on web-based nutrition for patients with cancer and cancer survivors. They were conducted in the United States, Australia, Korea, China, and in the United Kingdom, and were published between 2018 and 2022 in a variety of scientific journals. The number of participants ranged from 20 to 159.

**Conclusion:**

Web-based nutrition counselling helps cancer patients and survivors improve their dietary intake, impacts their weight and quality of life, and promotes a healthy lifestyle. Future research should evaluate (1) the differences in cost and coverage between face-to-face and web-based nutrition, (2) long-term outcomes, (3) cost-effectiveness, and last but not least, (4) the effectiveness of web-based nutrition in adolescents and children who suffer from cancer or who survived cancer, as nutritional status and body composition have a marked impact on clinical outcomes during and after treatment. The strength of this review lies in the large number of randomized controlled trials, which offer a guarantee of effectiveness and objectivity compared to cross-sectional studies.

## 1. Introduction

In 2018, the estimated cost of cancer treatment in Europe was 199 billion € (1). In 2020, 19,292,789 cases of cancer were reported worldwide, resulting in nearly 10 million (9,958,133) deaths (2).

GLOBOCAN estimates that by 2040, there will be 28.4 million new cancer cases annually, making cancer the leading cause of mortality and morbidity worldwide (3).

Cancer survivors need to be protected from long-term treatment-related side effects, such as new cancers, obesity, diabetes, osteoporosis, and cardiovascular diseases (4–7).

Against this background, nutrition education plays an important role, as obese adults with cancer have a poorer survival rate (8, 9), and obese children have been found to have increased chemotherapy toxicity, a higher relapse rate, and a lower overall survival rate than patients with normal body mass index (BMI) (10–12).

According to the findings of a recent meta-analysis that included 203 studies and more than 6.3 million participants, obesity is linked to a higher cancer death rate, particularly in patients with breast, colon, and uterine malignancies (13). Children and adult cancer survivors are among the patients who frequently neglect nutrition recommendations (4–7). Reduced food intake due to surgical, radiotherapy or medical interventions can lead to nutritional deficiencies such as malnutrition (14).

Sarcopenia and cachexia are typical issues in patients with cancer. The word “cachexia,” which derives from the Greek words “kakos”- meaning “bad,” and “hexis”- meaning “condition,” describes a disease that affects several organs and is associated with systemic disorders such as cancer (15). Systemic inflammation and a loss of body weight of at least 5% caused by severe waste of skeletal muscle and adipose tissue are its defining characteristics (15). In addition, the loss of skeletal muscle is thought to be a significant predictor of cancer risk, independent of BMI (15) and has been linked to a higher risk of chemotherapy toxicity, a faster tumor growth rate, subpar surgical outcomes, physical impairment, and shorter survival (16–18). The effectiveness of anti-cancer treatment often results in improvements in cachectic symptoms (19), while ineffective treatment may increase catabolism (20). Patients with advanced cancer, particularly those with lung or gastrointestinal diseases, often experience cachexia (21).

According to the European Working Group on Sarcopenia in Older People 2 (EWGSOP2), sarcopenia is a muscle disease (muscle weakness) caused by adverse muscle changes that develop throughout life and are more likely to lead to adverse outcomes such as falls, fractures, physical disability, and mortality (22–25). Regardless of disease stage, sarcopenia is a common occurrence that is correlated with age and antineoplastic therapy (25). While cancer cachexia is characterized by the loss of total body weight, sarcopenia is specific to the loss of lean muscle mass (26). The chronic underfunding of nutritional research on muscle wasting, sarcopenia, and cachexia has left many gaps and opportunities in this field.

In the age of new technologies and social media, the recent COVID-19 pandemic has enabled the use of telemedicine whenever possible and feasible. This includes using the Internet and technology to educate people about nutrition through discussion forums, blogs, exams, hyperlinks, chats and wikis (27). According to Ritterband et al. (28), the use of the Internet to deliver web-based interventions (WBI) or web-based learning (WBL) to patients is rapidly increasing and has therefore become an important part of the healthcare system, with important implications for the future of the system.

For example, during the COVID-19 pandemic, Lobascio et al. (29) suggested that massive efforts should be made to monitor nutritional status with the NUTritional RIsk AssessmENT (NUTRIENT) app for cancer patients at home, not only through regular phone and email contacts, but also through smartphone apps. In this review, we aimed to answer two questions: first, which groups of survivors and cancer patients are best suited to web-based nutrition interventions, and second, whether the use of web-based nutrition services is associated with health benefits for cancer survivors and patients in terms of physical activity, nutrition, and weight management. The aim of this article is to examine research findings on the effectiveness of weight management, healthy eating, and treatments delivered through web phone apps and/or the Internet as the main delivery channels in cancer patients or cancer survivors. The final section discusses the impact of web-based nutrition on quality of life.

## 2. Materials and methods

Publications on web-based nutrition in patients with cancer from all geographical regions were identified by systematically searching the PubMed database. This review was conducted in accordance with the Preferred Reporting Items for Systematic Reviews and Meta-Analysis (PRISMA) criteria for reporting and implementation (29). The Boolean operator “AND” was used to separate the following five categories of the search: (1) study design (e.g., intervention OR programme * OR study* OR clinical trial*), (2) intervention focus (e.g., weight loss OR diet* OR physical activity* OR efficacy*), (3) cancer (e.g., cancer OR neoplasm* OR malignancy*), (4) participant categories (e.g., survivors OR patients*), and (5) broad modality (email* OR web* OR internet* OR video conference* OR virtual visit*). Limits were used and non-human studies and papers in other languages than English were automatically excluded. Eligible articles satisfied the following inclusion criteria: clinical trials I-IV, clinical studies, controlled clinical trials, comparative studies, randomized controlled trials, and multicenter studies.

### 2.1. Data collection process

**Figure 1:**
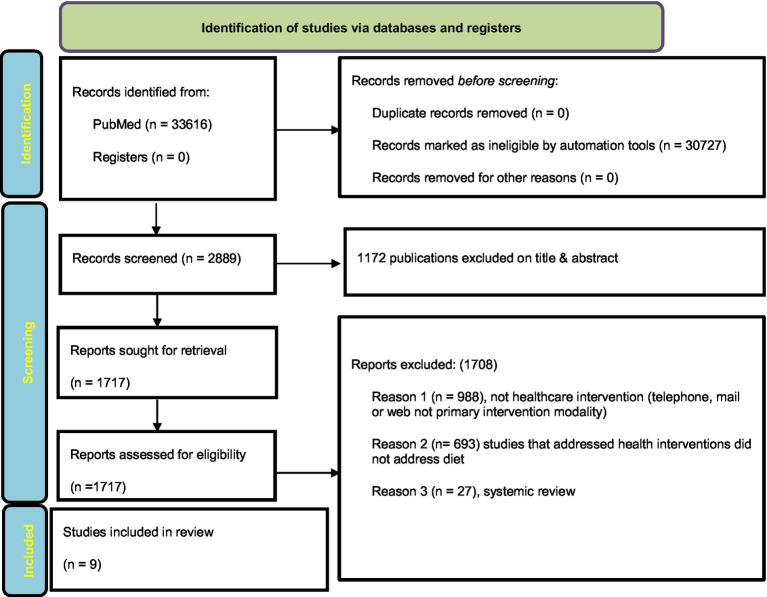
Diagram from PRISMA 2020 illustrating the process of choosing articles for review.

A total of 33.616 articles were found when the above search terms were entered into the PubMed database, along with the publication date, article types, and language filters. Finally, 9 articles that met the eligibility criteria were included in the systematic review. Figure 1 shows an overview of the selection process using the PRISMA 2020 flowchart in which the data were systematically extracted (30).

#### 2.1.1. Eligibility criteria

The following criteria were used for study inclusion: (a) the intervention aimed at changing dietary behavior, physical activity, and/or weight control (i.e., weight loss or weight maintenance) and these outcomes were reported; (b) the intervention targeted both children and adults with cancer who had either completed treatment or were undergoing treatment; (c) the intervention used telephone and/or the internet (including email and/or websites) to deliver the messages of the intervention.

Studies were excluded for the following reasons: (a) they were not available in English; (b) they included participants who received only palliative care; (c) they were available only as abstracts; (d) they did not contain sufficient information on the effectiveness of web-based nutrition on diet, physical activity, or weight loss; (d) review articles, systematic reviews, unpublished articles, dissertations, commentaries, abstracts of meetings and conferences, as well as case reports, book reviews, opinions, and editorials.

## 3. Results

The search approach resulted in the identification of a total of 33616 articles. However, the automation tools identified 30727 records as ineligible. After reading titles and abstracts, 1172 of the remaining 2889 articles were excluded, of which 1717 were selected according to the eligibility criteria. A further 1708 articles were eliminated as follows: (a) 988 studies were not health interventions (telephone, email, or Internet, not the primary intervention modality); (b) 693 studies were not related to diet; and (c) 27 were systemic reviews. Ultimately, 9 studies (31–39) met the inclusion criteria of our review and were subjected to data extraction.

### 3.1. Study characteristics

Table 1 presents the general characteristics of the studies included in this review (*n* = 9). From 2018 to 2022, articles were published in numerous scientific journals with different aims and objectives: *British Journal of Nutrition* (31), *Cancer Epidemiology, Biomarkers & Prevention* (32), *Nutrition and Cancer* (33), *JMIR mHealth and uHealth* (34), *Journal of Medical Internet Research* (35), *Journal of the Academy of Nutrition and Dietetics* (36), *Nutrition* (37), *JMIR Formative Research* (38) and *Nutrients* (39). All records were written in English.

**Table 1:** Details of the intervention and the results of the studies.

First author (ref) Country, year Link	Study population *N*/age/% female/%male Participation rate Recruitment	Intervention intensity/duration Comparison group(s) Delivery agent	Other intervention components	Effects of the intervention, including their sizes and significance
Mohamad et al. (31) UK, 2019 https://pubmed.ncbi.nlm.nih.gov/31177994/	Localized or locally advanced prostate cancer within the last 36 months and overweight or obesity, defined as BMI ≥ 25 kg/m^2^ *N* = 62/mean age = 65.5 years/100% male Medical oncologists and/or the personnel of an oncology clinic recruited candidates for the study	RCT A group meeting, a letter of support from their urology consultant, three phone consultations with a dietician scheduled 4 weeks apart, a pedometer, and access to online resources for diet and exercise are all included in the program. Usual care: diet and exercise support group	(a and b) Anthropometry (a and b) Quality of life (a and b) Acceptability and feasibility	12 weeks: The mean weight change of the mini-intervention group after 12 weeks was significantly different from the mean weight loss of the intervention group
Van Blarigan et al. (32) USA 2020 https://pubmed.ncbi.nlm.nih.gov/31941707/	Colorectal cancer survivors *N* = 50/median age = 55 years/66% female/34% male Recruited from cancer registries, oncology practices	RCT Group intervention (website, text messages) offers a simple diet tracking tool and a 12-week SMS programme Wait-list control	(a and b) Anthropometry (a and b) Dietary assessment (a and b) feasibility and acceptability	12 weeks: The intervention group consumed more whole grain products than the control group Systolic blood pressure seemed to decrease in the intervention group compared to the control group, which did not improve 24 weeks: The increase in whole grains in the intervention group appears to be maintained at 4 weeks On average, neither the participants’ weight nor their waistlines shrank during the study.
Hamilton-Reeves et al. (33) USA, 2021 https://www.ncbi.nlm.nih.gov/pmc/articles/PMC8371995/	Overweight men (BMI of 25 to 45 kg/m^2^) with localized prostate cancer who were scheduled for prostatectomy (robotic-assisted laparoscopic) had the option of either being treated (15) or not being treated (5) *N* = 20/ mean aged = 60.9 ± 5.7, 100% male Recruited in-person by ward personnel at two university clinics	RCT The nutrition intervention group received a weight maintenance phase: four support classes, weekly coaching by phone or email, tracking of diet and exercise Non-intervention group did not receive any nutrition or lifestyle counselling	(a and b) Anthropometrics and Vitals (a and b) Diet Adherence and Diet Quality (a and b) Tracking Physical Activity (a and b) Cardiometabolic Biomarkers (a and b) Quality of Life & Long-Term Outcomes (a and b) Feasibility	3 months follow-up: Short-term dietary and lifestyle adjustments, supported by coaching and self-tracking technologies, can positively alter biomarkers Only the intervention group experienced significant weight loss
Keum et al. (34) Korea, 2021 https://www.ncbi.nlm.nih.gov/pmc/articles/PMC8441607/	Patients with unresectable pancreatic ductal adenocarcinoma (PDAC) *N* = 40/mean age = 62–62, 5 years/37% female/63% male Recruited from cancer registries, physicians	RCT (a) The Noom users group offered the following interventions: (1) interactive interface with messages between coach and participant, (2) daily articles for basic health knowledge, (3) logging of food with color coding, and (4) automatic feedback on food choices (b) The group of non-Noom users did not receive a nutrition intervention, but only participated in the study assessments	(a and b) Quality of life (a and b) Skeletal muscle index (SMI)	12 weeks: All the study participants showed a significant improvement in the nutritional status according to the Patient-Generated Subjective Global Assessment (PG-SGA) score regardless of Noom app usage During chemotherapy, SMI decreased in both groups: in Noom users it decreased from 49.08 cm^2^/m^2^ to 46.08 cm^2^/m^2^ and in non-Noom users it decreased from 50.60 cm^2^/m^2^ to 42.97 cm^2^/m^2^. Although the decrease was greater in the non-Noom user group than in the Noom user group (−13.96% vs. -3.27%; *p* = 0.11), it was not statistically significant
Yang et al. (35) Korea, 2021 https://pubmed.ncbi.nlm.nih.gov/34448714/	Esophageal cancer patients receiving neoadjuvant chemoradiotherapy *N* = 38/mean age = 59 years, 63% male Recruited from cancer registries, oncology practices	Single arm The patients who used the mobile app (mHealth group) had the opportunity to record their caloric intake, physical activity and weight daily. In addition, the activity tracker in the app automatically counted the steps taken.	(a and b) Muscle loss (a and b) Malnutrition assessment (a and b) Laboratory markers (white blood cells, absolute neutrophil count, absolute lymphocyte count, neutrophil-to-lymphocyte ratio, platelet-to-lymphocyte ratio) (a and b) feasibility and effectiveness	8 weeks: The Prognostic Nutrition Index (PNI) decreased less thanks to the Health Care Mobile App, but could not stop the significant muscle loss. There was no discernible difference in the number of patients who suffered excessive muscle loss (SMI/50 days >10%) or in the change in SMI.
Terranova et al. (36) Australia, 2021 https://pubmed.ncbi.nlm.nih.gov/35182789/	Women aged 18 to 75 years with a body mass index of 25 to 45 kg/m^2^ who have been diagnosed with stage I to III breast cancer in the last 2 years *N* = 159/mean age = 55 ± 9 years/100% female Recruited by clinic staff at an oncology clinic and cancer centers	RCT Remotely delivered weight loss intervention Usual care	(a and b) Physical activity (a and b) Sitting time (a and b) Adherence to World Cancer Research Fund/American Institute for Cancer Research (WCRF/AICR) recommendations for cancer survivors	6-, 12-, and 18-months follow-up Participants randomly assigned to the intervention group aimed to lose 5 to 10% weight. The remote weight loss intervention resulted in some improvements in diet and physical activity, as well as sustained improvements in WCRF/AICR adherence scores.
Wang et al. (37) China, 2022 https://pubmed.ncbi.nlm.nih.gov/36183482/	Colorectal cancer survivors *N* = 60/mean age 69.07 ± 6,53 (intervention group), 67.50 ± 9.34 male 31/female 29 Recruited in-person by ward personnel	RCT (a) The intervention group received personalized nutritional interventions and telephone-based education through the WeChat app for 6 months (b) The routine care group received a follow up by telephone after 6 months	(a and b) Quality of life (a and b) Nutrition status (a and b) Dietary intake (a and b) The strength of the hands in kilograms	6 months follow-up: The patients’ subjective global rating was statistically lower in the nutrition group, and they consumed more energy and protein. The regular care group lost weight (0.00 kg; 95% confidence interval, −1.75 to 0.00), while the nutrition intervention group gained weight (2.00 kg; 95% confidence interval, 0.25–3.00). In terms of general health, physical role, emotional, cognitive and social functioning, the nutrition intervention group performed significantly better (*p* = 0.05).
Williams et al. (38) USA, 2022 https://pubmed.ncbi.nlm.nih.gov/35188468/	Adult survivors of cancers with > 80% 5-year survival *N* = 35/mean age = 62.1 years/54% female/46% male The following techniques were used to recruit participants: (1) identifying cancer patients from the University of Alabama at Birmingham Cancer Registry and sending an invitation; (2) getting in touch with community organizations and cancer survivor support groups in the area; (3) using local news advertisements; and (4) word-of-mouth	A website based on social cognition theory called SurvivorSHINE encourages cancer survivors to exercise, eat healthy and control their weight. The Godin Leisure-Time Exercise Questionnaire was used to assess physical activity subjectively, and ActiGraph accelerometers were used to assess it objectively. The Automated Self-Administered 24-Hour Dietary Assessment	Weight Time spent on the website Frequency of log-ins Page views	2 weeks: Total time spent on the website was positively related to increases in self-reported physical activity and physical activity measured with the accelerometer (*p* = 0.2) No associations were found between changes in healthy lifestyle knowledge, changes in body weight or food intake, or frequency of logins or total time spent on the website
Huggins et al. (39) Australia, 2022 https://www.mdpi.com/2072-6643/14/15/3234	Adults who had recently (within 4 weeks) been diagnosed with UGI cancer and were planning to start either surgery or medical (chemotherapy or radiation) treatment *N* = 111/mean age = 63, 2 years/37% female/63% male Recruited in person by surgeons and dietitians	Three group RCT Allocated to intervention *via* telephone Allocated to intervention *via* mobile APP Allocated to control	(a and b and c) Weight loss (a and b and c) Nutritional status (a and b and c) Survival rate	12-months: Although not substantially different, weight reduction throughout the course of the 12-month follow-up was less in the phone group compared to the mobile app group (*p* = 0.031) and compared to the control group (*p* = 0.075) The groups’ nutritional statuses were comparable.

#### 3.1.1. Study design and samples

Studies were conducted in the United States (*n* = 3), Australia (*n* = 2), Korea (*n* = 2), China (*n* = 1) and in the United Kingdom (*n* = 1). The number of study participants ranged from 20 to 159. Two studies had over 100, 6 studies had between 100 and 50 and 1 study had less than 50 participants. One study was published between 2018 and 2019, and the other 8 were published more recently (2020–2022).

Six of the included studies were randomized controlled trials (RCT) and only two studies had a single arm. Most studies (8 out of 9) targeted a single group of cancer survivors, namely survivors of breast cancer, prostate cancer, or colorectal cancer.

The identified web-based nutrition intervention studies (*n* = 2) were long-term studies (>6 months) and (*n* = 7) short-term studies (3–12 weeks) that examined the effects of nutrition counselling on a variety of nutrition-related outcomes (e.g., specific food intake, quality of life, body weight, or composition). None of the web-based studies compared face-to-face nutritional interventions in a group setting.

## 4. Discussion

Nutrition is a science (40) and a crucial component of care in the treatment of cancer at all stages, from diagnosis to survival, in 21^st^ century medicine, which we call predictive, preventative, and personalized (41). In the course of everyday clinical practice, which also involves coordinating food regimens for patients, the modern oncologist must deal with a variety of conflicting schools of thought that have gained prominence over the past 20 years (42–45).

On the one hand, we are aware that the requirements of cancer survivors are different from those of cancer patients; on the other hand, we know that malnutrition, sarcopenia, and cachexia are the main problems in the nutrition of cancer patients. With this in mind, clinical initiatives and the emphasis placed on enhancing treatment outcomes must logically incorporate a healthy diet and body composition.

The interventions studied in the articles were difficult to compare because they varied considerably in terms of the sample size, duration, study design, and cancer type. The results of web-based nutrition indicated that it was effective in achieving and maintaining dietary and anthropometric outcomes (including weight change). In the field of web-based nutrition, many tools have been used to assess the different variables of nutrition and body composition. The most common were dual-energy X-ray, abdominal computed tomography, height, body weight, body mass index, waist circumference, hip circumference, cardiometabolic biomarkers, and plasma levels of adiponectin, leptin, and resistin to name a few.

The discussion is divided into 4 sections. The first part examined the effectiveness of web-based nutrition in adolescent cancer survivors. The second section discusses the impact of web-based nutrition on adult cancer survivors. The third section discusses the impact of web-based nutrition on patients with cancer. The impact of web-based nutrition on quality of life is approached in the fourth section.

### 4.1. Web-based nutrition and its effectiveness in adolescent cancer survivors

Adolescent cancer survivors and patients are a particularly important target group for health promotion initiatives related to nutrition, as they often have unhealthy dietary habits (46) that could promote unwanted weight gain and possibly other chronic diseases as well as recurrences or secondary cancers. Keeping this in mind, we believe that the Internet could be a useful tool for nutritional counseling of children and adolescent who survived cancer, as they can now access it easily. Although considerable efforts have been made to develop nutritional risk screening and nutritional status assessment tools for children and adolescents with cancer (47), there was no single study to address web-based nutrition in adolescent cancer survivors.

### 4.2. The effects of web-based nutrition in adult cancer survivors

As the concept of long-term survivorship care intersects with many medical specialties, developing practice patterns that meet the needs of cancer survivors can be challenging. It is now well known that good nutrition and regular physical activity are important factors that can be modified in cancer survivors and are an essential part of follow-up care.

Wang et al. (37) showed that individual nutrition interventions *via* WeChat combined with telephone support from community health centers had a positive effect on weight and protein intake; general health; and physical, emotional, cognitive, and social functioning in colorectal cancer survivors. From this study, we can conclude that virtual health visits were of great benefit to participants. Moreover, this may be a long-term solution for comprehensive and personalized gastrointestinal cancer survivorship care and may retain patients at cancer centers.

Another study (31) comparing a 12-week healthy remote dietary intervention in colorectal cancer survivors showed that participants did not lose weight or waist circumference during the study, but had a higher intake of whole grain products and lower systolic blood pressure. This study suggests a possible anti-cancer and cardioprotective effect of web-based nutrition.

Williams et al. (38) conducted a single-arm pilot study of the Survivor SHINE lifestyle intervention website and found that increased use of the website correlated with improvements in physical activity. However, there was no association between the frequency of logins or total time on the website and improvements in healthy lifestyle knowledge or changes in body weight or food intake among survivors of cancers with >80% 5-year survival (e.g., breast, prostate, and thyroid cancers). It is important to note that cancer survivors who logged frequently and spent a lot of time on SurvivorSHINE were physically more active. In addition, this study also sheds light on the fact that non-Hispanic white female cancer survivors may be more engaged in web-based approaches. WeChat and the lifestyle intervention website SurvivorSHINE have different positive effects, and research shows that cancer survivors clearly show interest in web-based nutrition despite the different outcomes and benefits.

### 4.3. The impact of web-based nutrition in cancer patients

In theory, lifestyle changes such as diet and exercise in combination with chemotherapy can target tumor resistance mechanisms as a potential strategy to enhance treatment efficacy (44).

#### 4.3.1. Digestive tract cancer

In a three-arm study (38), two methods of health care delivered *via* telephone (synchronous) or *via* the internet-enabled mobile app “myPace” (random assignment) were directly compared with standard care in people with a recent diagnosis of upper gastrointestinal tract cancer made before the COVID -19 pandemic that triggered the rapid introduction of telehealth services. The findings imply that: (1) quality-adjusted life-years did not differ between the intervention group and the usual care group; (2) intensive nutrition counselling at a distance alone did not lead to adequate nutrition; and (3) service models other than face-to-face care allowed for the initiation of a nutrition intervention and contact with a dietitian at a much earlier stage. Although higher protein and energy intake has been achieved, we have learned that behavioral counselling alone is not sufficient to achieve adequate nutrition.

Yang et al. (35) highlighted in their study that in patients with esophageal cancer receiving neoadjuvant chemotherapy, an interactive mobile health coaching app that supported food self-care did not prevent excessive muscle wasting but promoted food self-care. The results of this study suggest that an individualized care model with appropriate physical activity and nutritional support may be needed to reduce muscle wasting and malnutrition.

Huggins et al. (39) found that the use of an interactive health coaching app as a nutrition and physical activity intervention produced similar negative results in terms of preventing muscle wasting in patients with esophageal cancer receiving neoadjuvant chemotherapy.

In summary, two studies (34, 38) that investigated muscle wasting in patients with esophageal cancer reached negative conclusions; however, one author (34) suggested that physical activity may improve outcomes. Only one study (33) presented positive results when using the Noon app. In addition, the study used psychological support, which we believe is useful in increasing motivation.

#### 4.3.2. Pancreatic cancer

The use of health education in patients undergoing chemotherapy for pancreatic ductal adenocarcinoma (PDAC) resulted in a remarkable improvement in their nutritional status, as shown by an objective measure (PG -SGAp score) by Keum et al. (34), regardless of whether the Noom app was used. Compared to non-Noom users, Noom users showed statistically significant improvements on the EORTC QLQ scales for global health status and quality of life. During chemotherapy, the skeletal muscle index (SMI) decreased in both groups. The non-Noom user group showed a greater decrease than the Noom user group, although the difference was not statistically significant. As these results show, mobile app-based coaching could help PDAC patients receiving chemotherapy to improve their nutritional and health status.

#### 4.3.3. Breast cancer

Terranova et al. (35) showed that telephone counseling sessions on diet and physical activity among women aged 18–75 years with a body mass index of 25–45 kg/m^2^ who had been diagnosed with stage I to III breast cancer in Australia in the previous 2 years had small to moderate effects on adherence to various behaviors measured by a composite score based on the 2007 WCRF/AICR guidelines but produced some improvement in diet and physical activity. The results of the study demonstrate the health benefits of programs targeting lifestyle behaviors that are consistent with recommendations for cancer survivors, as well as the potential for dissemination of such programs to women who have undergone treatment for early breast cancer.

#### 4.3.4. Prostate cancer

Although obesity is known to be associated with an increased risk of diagnosis of more aggressive forms of prostate cancer as well as prostate cancer recurrence and mortality, research on weight management in men with prostate cancer is relatively new and under-researched. Based on the assumption that physical activity in men with prostate cancer receiving androgen deprivation therapy may help prevent the loss of lean tissue, Mohamad et al. (31) conducted a study that showed modest weight loss but significant improvement in the quality of life in men treated for prostate cancer based on a self-help intervention that included diet and physical activity with dietary support for weight control. A study by Hamilton et al. (33), however, suggested that a weight management program tailored to men not only has an impact on weight in overweight men preparing for prostatectomy, but the results also suggest significant protective reductions in both visceral adiposity and leptin to adiponectin ratio, C-peptide, insulin, blood glucose levels, central adiposity, and systolic blood pressure through sessions focused on four components: lifestyle coaching, healthy eating with meal replacement, physical activity, and self-monitoring technology. Considering these results, we believe that this study is a good reproductive method for a large clinical trial in patients with prostate cancer.

### 4.4. Quality of life

Assessment of quality of life is a unique factor that is increasingly being considered when evaluating the nutrition of cancer survivors or patients. It reflects a patient’s current health status and can be used to assess the effectiveness of nutritional therapy. Perceptions of well-being across a range of dimensions, including symptoms (pain), physical abilities (mobility, strength), psychological states (anxiety, depression), and social isolation, can impact nutrition and determine the patient’s quality of life. There are several questionnaires for evaluating the quality of life, but it is not clear which one is the most appropriate. Some researchers (31, 33, 34, 37) have found it helpful to think of various measures of quality of life. They concluded that adequate nutritional status provided by web-based nutrition had a good outcome in terms of quality of life. However, future research is needed to replicate and consolidate these findings.

### 4.5. Feasibility

The studies (31–33, 35) assessed the feasibility and acceptability of the web-based nutrition intervention in different ways. Feasibility and acceptability were the primary outcomes in the study conducted by Van Blarigan et al. (32). The SMS program fulfilled the predetermined feasibility criteria (> 70% adherence) in this study, whereas the study website, which included a basic diet tracking tool, did not meet the criteria. Hamilton-Reeves et al. (33) demonstrated the feasibility of a weight management program customized for men to achieve a weight loss of ≥5% and a weight gain of ≤3% in overweight men preparing for prostatectomy.

### 4.6. How to evaluate the effectiveness and outcome of web-based nutritional support?

To evaluate the efficacy and outcomes of web-based nutritional support, we believe researchers should evaluate: (1) anthropometric data (body mass, fat-free mass, fat mass index, skeletal muscle), (2) biochemical data (albumin, proteins, carbohydrates, fats and glycaemia), (3) clinical data (risk of malnutrition, Crohn’s disease), and (4) nutritional data (dietary style of the individual patient). It is important to remember that certain cancer treatments, such as hormone therapy for breast and prostate cancer, may cause patients to gain weight.

### 4.7. The relevant role of patients’ associations

Patient organizations not only offer valuable services like peer mentoring, counselling, legal and financial support, but they can also encourage nutrition and physical activity by providing web-based nutritional resources, creating practical digital tools, and helping patients with the use of related applications (48).

## 5. Conclusion

Owing to its accessibility, web-based nutrition is a desirable supportive therapy for people who have been diagnosed with cancer or cancer survivors.

The last meta-review by Hanlon et al. (49) examined the efficacy of web-based nutrition in cancer patients or survivors and found limited research on the use of web-based nutrition, with results shown only in symptom scores. In our review, we can see an increasing interest in web-based nutrition, possibly due to the COVID-19 pandemic with improvements in anthropometric measurements, dietary changes, and quality of life.

This review had several *limitations*. First, there were differences in the study design. Second, several studies did not include a description of what the control group received during the intervention, demonstrating the need for thorough research reporting. Third, the ratio of men to women was not balanced because most studies were conducted on men, implying that future studies should target women. Fourth, not a single paper has examined the comparison between face-to-face nutrition counselling and a web-based nutrition intervention. We suggest that comparative studies be conducted to highlight the strengths and weaknesses of each strategy. Fifth, it would be helpful to examine which approach has a better cost–benefit ratio, given the differences in costs and coverage between face-to-face and web-based nutrition counselling. Sixth, children and adolescents with cancer or survivors are not a target group for web-based nutritional counselling, which represents a gap in our knowledge. Finally, important health problems, such as malnutrition and anorexia associated with cancer, have not been comprehensively assessed.

Nevertheless, there are also *strengths* of this review that should be highlighted. The first strength of the research papers is their attempts to offer an objective evaluation. Second, a panel of multidisciplinary experts including oncologists, psychologists, endocrinologists, and forensic scientists assessed the influence of web-based nutrition on cancer patients and survivors.

## Author contributions

BGI and DEL contributed to conceptualization and design. DEL, RP, and BH wrote the first draft, searched the database, read titles, and abstracts. BGI and VM supervised the elaboration of the manuscript. All authors wrote sections of the manuscript, selected articles according to the eligibility criteria, contributed to data extraction and manuscript revision, read, and approved the submitted version.

## Conflict of interest

The authors declare that the research was conducted in the absence of any commercial or financial relationships that could be construed as a potential conflict of interest.

## Publisher’s note

All claims expressed in this article are solely those of the authors and do not necessarily represent those of their affiliated organizations, or those of the publisher, the editors and the reviewers. Any product that may be evaluated in this article, or claim that may be made by its manufacturer, is not guaranteed or endorsed by the publisher.
